# Advances in biological functions and applications of apoptotic vesicles

**DOI:** 10.1186/s12964-023-01251-9

**Published:** 2023-09-25

**Authors:** Xianghui Zou, Qian Lei, Xinghong Luo, Jingyao Yin, Shuoling chen, Chunbo Hao, Liu Shiyu, Dandan Ma

**Affiliations:** 1 Department of Endodontics, Stomatological Hospital, School of Stomatology, Southern Medical University, No 366 Jiangnan Avenue South, Guangzhou, Guangdong Province 510280 China; 2 Department of Stomatology, Shenzhen Baoan Women’s and Children’s Hospital, Jinan University, Shenzhen, Guangdong Province China; 3 Hainan General Hospital (Hainan Affiliated Hospital of Hainan Medical University), Haikou, Hainan Province China; 4 State Key Laboratory of Military Stomatology & National Clinical Research Center for Oral Diseases & Shaanxi International Joint Research Center for Oral Diseases, Center for Tissue Engineering, School of Stomatology, The Fourth Military Medical University, 145West Changle Road, Xi’an, Shaanxi Province 710032 China

**Keywords:** Apoptotic vesicles, Subtypes, Direct therapeutic applications, Carriers, Vaccines, Diagnosis

## Abstract

**Background:**

Apoptotic vesicles are extracellular vesicles generated by apoptotic cells that were previously regarded as containing waste or harmful substances but are now thought to play an important role in signal transduction and homeostasis regulation.

**Methods:**

In the present review, we reviewed many articles published over the past decades on the subtypes and formation of apoptotic vesicles and the existing applications of these vesicles.

**Results:**

Apoptotic bodies were once regarded as vesicles released by apoptotic cells, however, apoptotic vesicles are now regarded to include apoptotic bodies, apoptotic microvesicles and apoptotic exosomes, which exhibit variation in terms of biogenesis, sizes and properties. Applications of apoptotic vesicles were first reported long ago, but such reports have been rarer than those of other extracellular vesicles. At present, apoptotic vesicles have been utilized mainly in four aspects, including in direct therapeutic applications, in their engineering as carriers, in their construction as vaccines and in their utilization in diagnosis.

**Conclusion:**

Building on a deeper understanding of their composition and characteristics, some studies have utilized apoptotic vesicles to treat diseases in more novel ways. However, their limitations for clinical translation, such as heterogeneity, have also emerged. In general, apoptotic vesicles have great application potential, but there are still many barriers to overcome in their investigation.

Video Abstract

**Supplementary Information:**

The online version contains supplementary material available at 10.1186/s12964-023-01251-9.

## Background

Cell apoptosis is a type of programmed cell death with specific morphological features, including cellular pyknosis, chromatin condensation, nuclear fragmentation, little or no alteration of organelles, and blebbing of the plasma membrane [1]. This physiological clearance mechanism is important in organismal development, tissue homeostasis and immune system function and participates in tumor regression [2–4]. The apoptosis process mainly consists of two stages. The first stage involves nuclear and cytoplasmic condensation, cell fragmentation and the formation of apoptotic products [2]. The second stage involves the engulfment or ingestion of apoptotic cells or products [2], termed “efferocytosis”, and occurs throughout the lifespan of an organism [3]. Efferocytosis can be conducted by professional phagocytes such as macrophages, non-professional phagocytes such as epithelial cells, and specialized phagocytes such as Sertoli cells [3, 5]. Professional phagocytes have the highest phagocytosis efficiency, and non-professional phagocytes also play an important role, especially when professional phagocytes are not sufficient or cannot easily access the apoptotic products [5]. Non-professional phagocytes can then produce cytokines [6, 7] as well as activate professional phagocytes [7, 8]. Similarly, professional phagocytes can also release cytokines to redirect the efferocytosis of non-professional phagocytes [9]. Various phagocytes can target and recognize apoptotic products through “find me” and “eat me” signals and ingest them through intracellular signalling [10].

Extracellular vesicles (EVs) are heterogeneous populations of lipid-bilayer membrane-bound vesicles that are derived from cells [11, 12]. After being discovered, they were considered “cytoplasmic waste” [12]. However, EVs play important roles in regulating the immune response, intercellular communication, and tissue homeostasis and participate in the growth and metastasis of tumors [12, 13]. EVs can mainly be classified into three categories: exosomes, microvesicles and apoptotic vesicles (ApoEVs) [14]. Owing to their advantages, including low immunogenicity, targeting capability to homologous cells, a long retention time and the ability to cross biological barriers [15, 16], EVs, especially exosomes, have been extensively explored since being discovered [17], while ApoEVs have received little attention. This is probably due to their disadvantages, such as relatively large and variable sizes, complicated contents, potential apoptosis-inducing abilities and easy clearance [16].

Although they have been explored less than other vesicles, ApoEVs have unique advantages. Apoptotic cells express or release “find me” signals, such as nucleotides, sphingosine-1-phosphate (S1P), lysophosphatidylcholine and fractalkine, to attract phagocytes, while presenting “eat me” signals, such as phosphatidylserine (PS), calreticulin, Annexin A1, and thrombospondin 1, to be recognized [18, 19]. As products from apoptotic cells, ApoEVs can be recruited and engulfed by immune cells, especially macrophages, and are therefore more suitable for immune cell-associated contexts or diseases [15, 20]. After being engulfed and ingested, they can suppress inflammation and are thus highly related to some inflammation-associated diseases [21]. In addition, ApoEVs can inherit information and substances from their parental cells and deliver them to recipient cells [22] while playing a critical role in signal transduction, homeostatic regulation [15] and tissue regeneration [23, 24].

Considering these advantages, ApoEVs have been utilized in four ways, namely, in direct therapeutic applications, as carriers, as vaccines and in diagnosis. However, as the contents and surface molecules of ApoEVs are rather complicated and their size and shape are rather variable, the heterogeneity of ApoEVs is a significant barrier to their further exploration and even use in the clinic. Initially, EVs produced by apoptotic cells were named apoptotic bodies (ApoBDs); however, from the present point of view, ApoBDs are ApoEVs that are approximately 1 μm to 5 μm in diameter [2, 25]. Apoptotic microvesicles (ApoMVs) and apoptotic exosomes (ApoExos) with smaller sizes were also found and characterized later [26–28]. The subtypes of ApoEVs differ from each other in terms of sizes, contents, properties and functions [28]. Besides, the types of source cells as well as induction and isolation methods can affect the subtypes of ApoEVs and the distribution of their contents and surface molecules. Thus, when new therapeutic functions of ApoEVs are found, the accurate production of exact ApoEV subtypes with the needed properties at scale is a challenge.

This review aims to clearly summarize the advances in applications of ApoEVs and their precise subtypes, induction methods and functional molecules to help overcome their heterogeneity and better translate them to the clinic. Additionally, we analysed the advantages and weaknesses of ApoEVs in various applications to determine whether the limitations of ApoEVs can be overcome by a deeper understanding and more effective utilization of ApoEVs or whether ApoEVs may not be suitable for applications in some fields. The review intends to analyse and address these problems.

### Subtypes and formation of apoptotic vesicles

#### Subtypes of apoptotic vesicles

During apoptosis, the cell membrane shrinks, separates, and packages the fragmented nucleus and cytoplasm, thus producing membrane-bound EVs named ApoBDs [29, 30]. ApoBDs were the first kind of ApoEVs characterized [2], and many studies still use the term “apoptotic bodies” to name EVs generated from apoptotic cells. Later, EVs derived from apoptotic cells with smaller sizes were also isolated and characterized. Thus, to be precise, ApoEVs include three subtypes whose sizes can overlap: ApoBDs (1-5 μm in diameter), ApoMVs (100-1000 nm in diameter) and ApoExos (<150 nm in diameter) [25, 28].

ApoBDs were first characterized by Kerr et al. [2] and used to be regarded as cell debris [31]. As they are relatively large, they are easy to be detected by light and electron microscopy [28]. Although commonly considered 1-5 μm, the size of ApoBDs can be influenced by the type and size of their parental cells as well as their formation mechanism [28]. For example, the diameter of ApoBDs from human Jurkat T cells, LIM1215 colon carcinoma cells and THP-1 monocytic cells can reach 8–10 μm [28]. And many beaded apoptopodia of apoptotic THP-1 monocytic cells have a diameter less than 1 μm [28]. Thus, it is not accurate to distinguish subtypes of ApoEVs based only on size [28]; instead, size, morphology by electron microscopy and biogenesis should all be considered [32].

Smaller ApoEVs with sizes of approximately 0.1-1 μm in diameter, which are similar to microvesicles released by viable cells, are named ApoMVs [25, 28]. ApoBDs from stress-activated apoptotic human endothelial cells (ECs) contain histones, while ApoMVs from the same cells contain few histones [33]. In addition, ApoBDs can cause sterile inflammation, while ApoMVs cannot [33]. However, membrane microparticles from polymorphonuclear cells can stimulate plasmacytoid dendritic cells (DCs) to secrete interferon (IFN) α via DNA in vesicles [34] while ApoMVs from infected cells can stimulate CD8^+^ T cells [35].

Regarding ApoExos, after activation of caspase, apoptotic ECs release exosome-like nanovesicles, which express exosomal markers, translationally controlled tumor protein (TCTP) and tumor susceptibility gene 101, and have irregular shapes and similar sizes as exosomes [32]. Besides, TCTP has also been detected in multivesicular bodies of apoptotic ECs [32]. Chen et al. isolated ApoEVs from mouse thymocytes and proved that they have a size corresponding to exosomes [36]. Dieudé et al. also isolated and characterized apoptotic exosome-like vesicles from ECs with diameters of 30-100 nm [27]. These vesicles expressed many exosome protein markers but did not express some classical exosome markers, suggesting that they were similar to but not the same as exosomes in terms of the proteome [27]. In addition, the proteomes of these vesicles and ApoBDs from the same cells were distinct, suggesting differences in their formation mechanisms and protein sorting circumstances [27]. Ribosomal, cytosolic, nuclear, and mitochondrial proteins and proteins from the endoplasmic reticulum were abundant in ApoBDs, while basement membrane proteins, extracellular matrix, and lysosomal proteins were abundant in ApoExos [27]. Additionally, proteins specific to exosome-like vesicles mainly contribute to proteasomal degradation and ligase activities, and those of ApoBDs mainly function in coping with RNA and targeting abilities [27]. ApoExos enhanced the generation of autoantibodies and allograft rejection, while ApoBDs did not [27]. Compared with ApoBDs released by apoptotic ECs, only ApoExos were proven to have immunogenicity in mice [37]. Park et al. segregated apoptotic exosome-like vesicles, which had a size, density, morphology, and protein expression profile similar to those of exosomes from viable cells [38]. However, they possessed specific marker proteins, sphingosine-1-phosphate receptors 1 and 3 (S1PR1 and 3) [38]. Although the traditional theory suggests that apoptosis is a process that does not lead to inflammation, by releasing damage-associated patterns (DAMPs), apoptosis can cause immune responses [38]. ApoExos may serve as DAMPs [38].

During the separation and extraction process, subtypes of ApoEVs cannot be clearly distinguished [28]. Except for size, there are no other standardized and executable criteria to distinguish among various ApoEVs [39]. In some experiments, the vesicles utilized do not match the corresponding sizes. For example, Wang et al. isolated ApoEVs that were approximately 100–1000 nm in diameter and called them small apoptotic bodies [16]. Various subtypes of ApoEVs not only vary in size but also contain different contents and surface proteins and have different characteristics [28]. Thus, distinguishing subtypes of ApoEVs with criteria in addition to size and stably obtaining vesicles with similar contents and properties is challenging.

#### Formation of apoptotic vesicles

The formation mechanisms of different subtypes of ApoEVs also vary. The formation of ApoBDs is tightly controlled [40] and caspase-mediated [41]. For example, overexpression of Bcl-2 can suppress the formation of ApoBDs by inhibiting caspases [42]. Moreover, adenosine diphosphate (ADP)-ribose polymers are essential for the formation of ApoBDs in HL-60 cells [43], while suppressing mono-ADP-ribosyltransferase activity can inhibit ApoBD formation [44]. Functional microtubules and myosin light chain kinase (MLCK) play roles during the process of nuclear shrinkage and loading of nuclear material into apoptotic cell-derived membranous vesicles respectively [45]. The fungal metabolite cytochalasin B can inhibit the formation of ApoBDs by suppressing actin polymerization, which proves the importance of microfilament assembly for the formation of ApoBDs [46]. In Jurkat T cells, either inhibition of the plasma membrane channel pannexin 1 (PANX1) or suppression of Rho-associated protein kinase 1 (ROCK1) and PANX1 can promote the formation of ApoBDs [47]. The former functions by enhancing the separation of membrane blebs through apoptopodia, and the latter functions by producing beaded apoptopodia [47].

The process of ApoBD formation can be divided into three stages: plasma membrane blebbing, formation of thin membrane protrusion and distinct ApoBD generation [28]. During the process of apoptotic body formation, according to the cell's volume-to-surface ratio, a cell membrane surface increase or a cell volume decrease must occur [48]. The apoptotic membrane blebbing step is modulated by several protein kinases, including ROCK1, MLCK, LIM domain kinase 1 (LIMK1), and p21-activated kinase (PAK2) [40, 49]. Tixeira et al. demonstrated that ROCK1 but not LIMK1 or PAK2 is a key regulator of apoptotic membrane blebbing [49]. Regarding apoptotic membrane protrusions, three kinds of protrusions can be formed: microtubule spikes, apoptopodia and beaded apoptopodia [50–52]. Microtubule spikes are rigid membrane protrusions that are rich in microtubules and can contribute to the formation of ApoBDs even without a membrane blebbing step [50]. The second kind of membrane protrusion, apoptopodia, is thin and string-like, and the caspase-activated PANX1 channel is a negative regulator of their formation [40, 52]. It was suggested that PANX1 is also a negative regulator of nuclear content packaging and can regulate ApoBD size [53]. Apoptopodia can be formed without actin polymerization or microtubule assembly [52]. The last kind of protrusion, beaded apoptopodia, is a string of connected membrane vesicles, the diameter of which is mainly 1-3 μm [51]. They can also be formed without membrane blebbing and are modulated by caspase-activated PANX1 channels and vesicular transport [51]. Many more ApoBDs can be formed after the initial formation of beaded apoptopodia [51]. Vesicular trafficking positively modulates the generation of beaded apoptopodia, while the membrane channel pannexin 1 negatively modulates it [51].

For ApoMVs and ApoExos, the formation mechanisms are different. Similar to microvesicles produced by viable cells, apoptotic microvesicles or microparticles are formed by budding of the apoptotic cell membrane [54]. However, their formation mechanism is not fully understood. Similarly, as exosomes are generated through multivesicular body fusion with the plasma membrane and subsequent release [55], the production of ApoExos resembles this process [32] and is related to the S1P-S1PR signalling pathway [38]. By comparing the protein expression of ApoExos and conventional exosomes from viable cells, it was found that the generation of ApoExos may not be dependent on the endosomal-sorting complexes required for the transport pathway but on S1P–S1PR signalling [38]. In addition, the activation of caspase-3 may also be important for the release of ApoExos, and the different downstream targets of caspase-3 lead to the generation of ApoBDs or ApoExos [27]. Large autolysosomes are a site of ApoExo biogenesis, while caspase-3 can modulate autolysosome cell membrane interactions [56].

During the formation process of ApoEVs, an important characteristic is the exposure of PS. PS is an anionic cellular phospholipid that transitions from the inner leaflet to the outer leaflet of the cell membrane when the cell undergoes apoptosis [20, 57]. The externalization of PS depends on caspase, while flippase is inactivated and scramblase is activated at the cytomembrane during the process [58]. Caspase cleavage at caspase recognition sites leads to deactivation of flippase, which is necessary for PS exposure [58]. As the most well-characterized “eat me” signal of ApoEVs, PS is recognized by phagocytes, especially macrophages, to clear apoptotic cells [20, 59]. After being recognized through PS, apoptotic vesicle membranes can be engulfed, and this process activates the anti-inflammatory response [15, 57, 60].

Apoptotic stimuli do not influence the distribution of DNA in ApoEVs [47]. For example, Li et al. induced adipose mesenchymal stem cells (ADSCs) to undergo apoptosis with staurosporine (STS), desacetylcinobufotalin, hydroxyurea, or hypocrellin B and found that the levels of microRNA (miR)-21–5p, an important miRNA that can regulate inflammation, were increased in all ApoEVs produced via the above approaches [61]. The contents of ApoEVs can be influenced by their formation mechanism, and the type of parental cells affects their formation mechanism [47]. For example, ApoEVs from Jurkat T cells contain more DNA, while ApoEVs from THP-1 monocytes contain more mitochondria [47]. Additionally, by comparing ApoEVs from human embryonic stem cells (ESCs) and human umbilical cord mesenchymal stem cells (UCMSCs), investigators found that ApoEVs from ESCs inherit more nucleoprotein from donor cells, while ApoEVs from these two kinds of cells differ in size [62]. ApoEVs from UCMSCs have a larger diameter and more protein per vesicle, while ESCs produce more ApoEVs [62]. But ApoEVs from ESCs and induced pluripotent stem cells (iPSCs) are similar in size, protein level, and protein distribution [62].

However, apoptotic stimuli may influence the total production of ApoEVs [62]. ESCs incubated under serum-free conditions produced more ApoEVs than those incubated with STS, and ApoEVs produced by these two approaches both expressed exosome markers as well as the ApoEV markers calreticulin and cleaved caspase-3 [62]. In addition, they both expressed pluripotency molecules inherited from ESCs [62].

### Existing applications of apoptotic vesicles

#### Direct therapeutic applications of apoptotic vesicles

##### Apoptotic vesicles from mesenchymal stem cells

Mesenchymal stem cell transplantation has been shown to be effective in many animal experiments and even clinical trials [63, 64]. However, they face challenges including ethical and safety problems, immunoreactivity, limited retention rates, and restricted cell sources; thus, EVs from mesenchymal stem cells (MSCs), which inherit various bioactive molecules from source cells, may be superior for treating diseases [23, 61]. Stem cells have the abilities of self-renewal and multipotent differentiation, and sometimes they exert their best effects after apoptosis, as only a small number of MSCs can survive after transplantation [23, 63]. Besides, ApoEVs play an important role in intercellular communication between stem cells and neighbouring cells. For instance, adjacent stem cells take up ApoBDs from dying stem cells and are stimulated to proliferate more actively through Wnt signalling pathways [65]. In fact, ApoEVs from various kinds of MSCs have been demonstrated to have direct therapeutic effects in vivo and in vitro [23–25, 61–63, 66–70].

Early in 2018, Liu et al. demonstrated the importance of ApoBDs to MSCs [66]. Specifically, the proliferation and differentiation capabilities of bone marrow mesenchymal stem cells (BMMSCs) decreased with a reduction in apoptotic body formation [66]. However, after infusing exogenous ApoBDs, MSCs could take up ApoBDs through integrin αvβ3 and ultimately ubiquitin ligase RNF146 and miR-328-3p to suppress Axin1, thus activating the Wnt/β-catenin pathway and impairing the abilities of MSCs [66]. In vivo, exogenous ApoBDs could also improve the osteopenia phenotype, whether the phenotype is related to apoptosis or not [66]. Thus, it can be hypothesized that ApoEVs also regulate other apoptosis-related pathological symptoms. Later, the effects of ApoEVs from BMMSCs were demonstrated in a myocardial infarction model [63]. After transplantation, MSCs underwent apoptosis and released ApoBDs, which activated transcription factor EB (TFEB)-dependent lysosome biogenesis and thus promoted autophagy in recipient ECs [63]. Subsequently, the induction of autophagy could promote angiogenesis through the AKT-mediated vascular endothelial growth factor signalling pathway, increase nitric oxide levels and ameliorate myocardial infarction [63]. This research provides strategies to use ApoEVs from MSCs to promote vascularization. Focusing on the challenges of angiogenesis and apoptosis of donor MSCs in an ischaemic-hypoxic environment, the roles of ApoEVs from human dental pulp cells (hDPSCs) in angiogenesis in the dental pulp were explored [24]. ApoEVs from hDPSCs could be internalized by ECs, transfer the mitochondrial Tu translation elongation factor to promote autophagy of ECs, which was also dependent on TFEB, and enhance angiogenesis [24]. In other ischaemic-hypoxic tissues or environments in which cells are susceptible to apoptosis, apoptotic cells may also transmit molecular messages in ApoEVs to modulate organismal homeostasis and contribute to pathological processes.

Additionally, ApoEVs from MSCs can influence macrophages. ApoEVs from BMMSCs were proven to promote macrophage polarization towards the M2 phenotype, which can enhance the function of fibroblasts, ultimately facilitating cutaneous wound healing [23]. Later, it was found that ApoBDs from ADSCs can also promote wound healing by increasing miR-21-5p in macrophages to target Krüppel-like factor 6, which can promote the formation of blood vessels and the migration of fibroblasts [61]. Similar regenerative effects were also observed in the reproductive system. Xin et al. added ApoBDs from MSCs into a hyaluronic acid hydrogel to promote endometrial regeneration and suppress fibrosis [67]. By activating mitochondrial bioenergetics, the proliferation and angiogenesis of macrophages, human endometrial stromal cells, and ECs were promoted, and inflammation was modulated [67]. The therapeutic effect of ApoBDs was proven in acute endometrial damage and intrauterine adhesion models [67]. This study provides a strategy to overcome the low retention and engraftment properties of ApoEVs and realize their in situ delivery [67].

The function of ApoEVs derived from pluripotent stem cells was also studied. ApoEVs from ESCs and iPSCs have similar abilities to enhance wound healing and are both better than ApoEVs from human UCMSCs [62]. ESCs produce fewer exosomes than ApoEVs, which can inherit pluripotent molecules from their source cells [62]. ESC-derived ApoEVs can better regulate the stemness of mouse skin MSCs, promote their proliferation and osteogenic differentiation, suppress their adipogenic differentiation, and enhance cutaneous healing via the SOX2/Hippo signalling pathway [62]. If ApoEVs from pluripotent stem cells can be investigated more thoroughly and applied in the clinic, they may have the potential to be substitutes for their parental cells to avoid their disadvantages, including potential oncogenicity and immunogenicity [62]. Compared with MSCs from other sources, pluripotent stem cell-derived MSCs have improved stability and stemness [62].

By investigating the distribution of ApoEVs from MSCs after intravenous infusion, Ma et al. found that exogenous ApoEVs can be metabolized in the integumentary system and promote cutaneous wound healing and hair regeneration [69]. Small extracellular vesicles (sEVs) from the same parental cells can enhance cutaneous wound healing but cannot promote hair regeneration, which may be due to the different contents of ApoEVs and sEVs [69]. The Wnt/β-catenin pathway and mechanical force participate in this process [69]. Furthermore, via the interaction between electrostatic charge and neutrophil extracellular traps, exogenous human bone marrow MSC-derived ApoEVs can accumulate in the bone marrow of septic mice [68]. They can alleviate multiple organ dysfunction and increase the survival rate of septic mice by converting neutrophil NETosis to apoptosis through the Fas/Fas ligand (FasL)/reactive oxygen species (ROS) pathway [68]. Thus, altering the death pattern of immune cells to regulate inflammation may be a promising therapeutic strategy [68]. In addition, further studies tracing the fate and functions of ApoEVs in these organs may elucidate the roles of ApoEVs in the human body more thoroughly.

In the above studies, ApoEVs from MSCs were mainly used in the regeneration of tissues such as bone, cardiac muscle, skin, hair follicles, the endometrium and dental pulp. Whether the regeneration of other tissues can also benefit from ApoEVs derived from MSCs and the common features of these tissues need more exploration.

The above studies mainly utilized the regeneration-promoting characteristics of MSCs, and there are studies on the ability of ApoEVs from MSCs to induce apoptosis and regulate inflammation. Wang et al. found that ApoEVs from MSCs can cause the apoptosis of multiple myeloma cells and improve symptoms [70]. Specifically, ApoEVs induce the influx of Ca^2+^ into tumor cells to enhance Fas transport from the cytoplasm to the cell membrane while expressing FasL to activate the Fas/FasL/ROS pathway [70]. For immunomodulation, in addition to ameliorating sepsis, Zheng et al. proved that ApoEVs derived from BMMSCs can reestablish liver macrophage homeostasis and thus possess therapeutic potential for T2D by inhibiting diseased liver macrophage infiltration and activation through the efferocytosis of ApoEVs by macrophages via calreticulin [25]. These studies suggest the potential of ApoEVs in treating other tumors and immune-related diseases.

##### Apoptotic vesicles from immune cells

Cells upregulate “eat me” signals when undergoing apoptosis, and apoptotic cells can be quickly engulfed by phagocytes in response to these signals [60, 71]. After being recognized by macrophages or other phagocytes, apoptotic vesicle membranes can be engulfed, and the anti-inflammatory response can be activated [15, 57, 60]. This process induces anti-inflammatory activities by shifting the phenotype of macrophages from M1, proinflammatory macrophages, to M2, anti-inflammatory macrophages [21]. Thus, during this process, ApoEVs, especially those from immune cells, are ideal EVs to regulate inflammation [15].

Through efferocytosis, engineered ApoBDs from activated T cells and neutrophils can regulate inflammation in myocardial infarction, colitis and cutaneous healing [15, 72]. ApoEVs from thymocytes can also increase the expression of TGFβ in macrophages through PS and the transcription factor FOXO3 to regulate inflammation in contexts such as colitis [36]. Additionally, Yang et al. constructed T-cell-depleting nanoparticles that can recruit activated T cells and induce their apoptosis, promoting the production of endogenous ApoEVs that can rescue impaired MSCs [73]. Through apoptotic signals from the ApoEVs produced via this process, macrophages can regulate the Treg/Th17-cell balance and improve the osteopenia phenotype [73].

Interestingly, ApoEVs also contribute to the influence of macrophages on MSCs. Zhu et al. found that ApoEVs from RAW 264.7 macrophages enhance adipogenesis and inhibit osteogenesis of human ADSCs in vitro and in vivo through miR155 in vesicles by targeting the SMAD2 signalling pathway [74]. However, whether ApoEVs from macrophages modulate the osteogenesis and adipogenesis of other kinds of MSCs in a similar way needs further investigation. Moreover, the effect of ApoEVs from polarized macrophages on MSCs has not yet been explored.

##### Apoptotic vesicles from other cells

In other cell types, ApoEVs also transfer important molecules from apoptotic cells to live cells. Although different source cells can differentiate from the same progenitor cells, ApoBDs from bone marrow macrophages, preosteoclasts, and mature osteoclasts (mOCs) have different functions in bone remodelling [29, 75, 76]. ApoBDs inherit the characteristics of their parental cells, such as the ability of preosteoclast apoptotic bodies (pOC-ABs) to enhance endothelial progenitor cell proliferation and differentiation and the ability of mature osteoclast apoptotic bodies (mOC-ABs) to promote osteogenic differentiation [29]. In addition, compared with microvesicles and exosomes derived from osteoclasts at different developmental stages, ApoBDs from mOCs have the best osteogenic effect [76]. Together, these two kinds of ApoBDs can facilitate bone defect healing [29]. mOC-ABs exert the strongest osteogenic effects among vesicles from these cell types by activating receptor activator of NF-κB ligand reverse signalling [76], while pOC-ABs promote endothelial progenitor cell differentiation via platelet-derived growth factor-BB through the PI3K/AKT pathway [75]. Specific lncRNAs in different kinds of ApoBDs also contribute to vesicle functions [29], which can be utilized in bone remodelling and the treatment of primary and metastatic bone tumors [29, 75].

During atherosclerosis, endothelial cell-derived ApoBDs are produced [77]. Containing abundant miR-126, these vesicles can increase the production of CXCL12 in ECs and thus promote a feedback loop via its receptor CXCR4, which can recruit progenitor cells and stabilize atherosclerotic plaques [77]. When incubated with ApoBDs from mature ECs, endothelial progenitor cells (EPCs) can engulf ApoBDs, and the proliferation and differentiation abilities of EPCs are then increased, demonstrating extracellular communication between progenitor cells and damaged somatic cells [78].

**Fig. 1:**
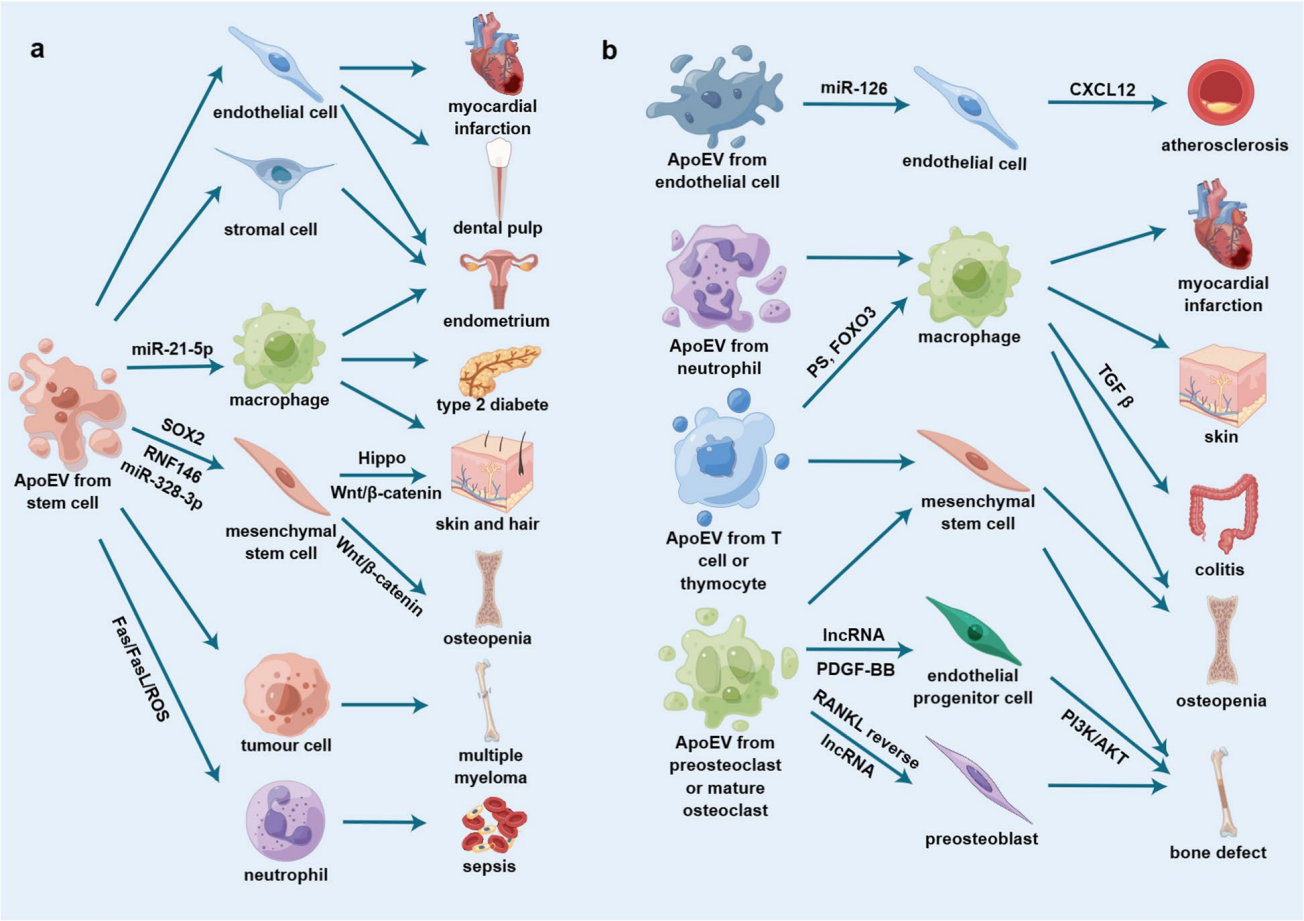
Direct therapeutic applications of ApoEVs (**a**) ApoEVs from stem cells; (**b**) ApoEVs from other cells. ApoEVs from various types of cells (especially stem cells) have been utilized and can function on specific recipient cells to address multiple types of diseases through specific signalling molecules or pathways. (Figure was created using Figdraw). Abbreviations: ApoEVs, apoptotic vesicles; miR, microRNA; FasL, Fas ligand; ROS, reactive oxygen species; PS, phosphatidylserine; TGF β, transforming growth factor-β; lncRNA, long non-coding RNA; PDGF, platelet derived growth factor; RANKL, receptor activator of nuclear factor κB ligand

As the isolation methods and the sizes of ApoEVs used in different experiments vary, whether these factors influence the contents and characteristics of ApoEVs needs to be verified to consistently produce the desired ApoEVs. Thus, we summarized the direct therapeutic applications of ApoEVs from various kinds of cells and their relevant details (Table 1 and Fig. 1).

**Table 1:** Direct therapeutic applications of apoptotic vesicles

Published year	Term	Parental cell	Size of vesicles	Induction method	Isolation method	Function	Target cell	Animal	Applied method	Disease model	Ref.
2022	Apoptotic vesicles	RAW264.7 macrophages	240.6±115nm	STS and serum-free	Sequential centrifugation	Inhibit osteogenesis and promot adipogenesis of MSCs	Human ADSCs	Nude mice	Implant with collagen sponges or β-TCP	N/A	[74]
2022	Apoptotic vesicles	Human BMMSCs	150.1±14.8nm	STS	Sequential centrifugation	Switch neutrophils NETosis to apoptosis, ameliorate multiple organ dysfunction and improve survival in septic mice	Bone marrow neutrophils	Mice	Inject intravenously	Sepsis	[68]
2022	Apoptotic vesicles	Murine BMMSCs	Approximately 50-1000nm	Serum-free and STS, or DCPy with ultralow-power light irradiation	Sequential centrifugation followed by sequential filtering analysis	Promote wound healing and hair growth via activation of Wnt/β-catenin pathway	Skin and hair follicle MSCs	Mice	Inject subcutaneously or intravenously	SLE	[69]
2022	Apoptotic vesicles	Human DPSCs	Around 100-800nm	STS	Sequential centrifugation	Transport TUFM to activate ECs autophagy and promote ECs angiogenesis via the TFEB-induced autophagy-lysosome pathway	ECs	Nude mice, beagle dogs	Implant tooth scaffolds filled with ApoEVs subcutaneously in the dorsum(mice), inject ApoEVs gel material into the root canal of the anterior tooth after removal of the pulp(dogs)	N/A	[24]
2022	Apoptotic vesicles	Human ESCs and iPSCs	Approximately 50-200nm	STS and serum-free	Sequential centrifugation	Promote mouse skin wound healing via transferring SOX2 into skin MSCs to activate Hippo signaling pathway	Skin MSCs	Mice	Inject intravenously	Skin wound	[62]
2022	Apoptotic bodies	Mouse ADSCs	800-1600nm	STS, desacetylcinobufotalin, hydroxyurea, or hypocrellin B	Sequential centrifugation	Induce M2 polarization of macrophages	Macrophages	Mice	Inject subcutaneously	Skin wound	[61]
2021	Apoptotic extracellular vesicles	Mouse T cells	200-2000nm	T cell-depleting nanoparticles with Fas-ligand	Sequential centrifugation	Promote macrophages transformation towards the M2 phenotype	Macrophages, BMMSCs	Mice	Induce ApoEVs production in vivo	Osteoporosis	[73]
2021	Apoptotic bodies	Human UCMSCs	200-3000nm	UVC light	Sequential centrifugation	Induce macrophages immunomodulation, cell proliferation, and angiogenesis	Macrophages, human endometrial stromal cells, ECs	Mice, rats	Load into a hyaluronic acid hydrogel and inject in situ	Acute endometrial damage (mice), intrauterine adhesions (rat)	[67]
2021	Apoptotic bodies	Rat bone marrow neutrophils	800-1200nm (Neu-ABs), 100-400nm (eNABs)	STS	Sequential centrifugation	Inflammation-tropism and immunoregulatory effects	Macrophages	Rats	Inject intravenously	Myocardial infarction	[72]
2021	Apoptotic vesicles	Human BMMSCs	<700nm	STS	Sequential centrifugation	Alleviate macrophages infiltration and promote macrophages polarization towards M2 phenotype	Macrophages	mice	Inject intravenously	Type 2 diabetes	[25]
2021	Apoptotic extracellular vesicles	Mouse BMMSCs	Around 50-250nm	STS and serum-free	Sequential centrifugation	Facilitate Fas trafficking from the cytoplasm to the cell membrane of tumor cells by evoking Ca2+ influx and elevating cytosolic Ca2+, use Fas ligand to activate the Fas-FasL pathway	Multiple myeloma cells	Mice	Inject intravenously	Multiple myeloma	[70]
2021	Apoptotic bodies	Mouse pOCs and mOCs	Unknown	STS	Sequential centrifugation	pOC-ABs induce EPCs differentiation and increase ECs formation, mOC-ABs induce osteogenic differentiation of MSCs and facilitate osteogenesis	EPCs, MSCs	Mice	Graft decalcified bone matrix pre-incubated with different ApoBDs in the defect area	Bone defect	[75]
2020	Apoptotic bodies	Murine BMMSCs	Approximately 600-1600nm	STS	Sequential centrifugation	Trigger the polarization of macrophages towards M2 phenotype	Macrophages (macrophages further enhance the migration and proliferation abilities of fibroblasts)	Mice	Locally administrate in skin wound	Skin wound	[23]
2020	Apoptotic bodies	Mouse T cells	700-2000nm (ABs), 100-600nm (cABs)	STS	Sequential centrifugation	Target inflammatory regions and modulate inflammatory processes	Macrophages	Mice	Inject intravenously	Cutaneous inflammatory wound, colitis	[15]
2020	Apoptotic bodies	Mouse pOCs and mOCs	Approximately 1-4μm	STS	Sequential centrifugation and sequential filtering	pOC-ABs induce angiogenesis, mOC-ABs promote osteogenesis	EPCs, MC3T3-E1	Mice	Graft decalcified bone matrix pre-incubated with different ApoBDs in the defect area	Bone defect	[29]
2020	Apoptotic bodies	Mouse and rat BMMSCs	400-700nm	STS	Sequential centrifugation	Enhance angiogenesis of ECs and improve cardiac functional recovery	ECs	Rats	Inject intramyocardially	Myocardial infarction	[63]
2019	Apoptotic extracellular vesicles	Mouse thymocytes, Jurkat cells	50-100nm	Gamma ray or UV irradiation	Sequential centrifugation	Promote TGFβ production in macrophages	Macrophages	Mice	Inject intraperitoneally	Colitis	[36]
2019	Apoptotic bodies	mOCs	Approximately 1-4μm	Nitrogen-containing bisphosphonate alendronate	Sequential centrifugation and sequential filtration	Promote osteogenic differentiation	MC3T3-E1	N/A	N/A	N/A	[76]
2018	Apoptotic bodies	BMMSCs	1-5μm	STS	Sequential centrifugation followed by sequential filtering	Maintain MSCs homeostasis and ameliorate osteopenia	BMMSCs	Mice	Inject intravenously	Osteopenia	[66]
2009	Apoptotic bodies	Vascular ECs	Unknown	Serum and growth factors-free	Sequential centrifugation	Convey paracrine alarm signals to recipient vascular cells that trigger the production of CXCL12	Vascular ECs	Mice	Inject intravenously	Atherosclerosis	[77]

*Abbreviations: STS* Staurosporine, *MSCs* Mesenchymal stem cells, *ADSCs* Adipose-derived stem cells, *β-TCP* β-tricalcium phosphate, *BMMSCs* Bone marrow mesenchymal stem cells, *SLE* Systemic lupus erythematosus, *DPSC* Dental pulp stem cells, *TUFM* Mitochondrial Tu translation elongation factor, *ECs* Endothelial cells, *TFEB* Transcription factor EB, *ESCs* Embryonic stem cells, *iPSCs* induced pluripotent stem cells, *UCMSCs* Umbilical cord-derived mesenchymal stem cells, *UV* Ultraviolet, *Neu-ABs* Apoptotic bodies from neutrophils, *eNABs* engineered neutrophil apoptotic bodies, *pOCs* Preosteoclasts, *mOCs* mature osteoclasts, *pOC-ABs* preosteoclast apoptotic bodies, *mOC-ABs* mature osteoclast apoptotic bodies, *EPCs* Endothelial progenitor cells, *ABs* Apoptotic bodies, *cABs* chimeric apoptotic bodies

##### Apoptotic vesicle-mimicking nanoparticles

Some investigators did not isolate ApoEVs from cells; instead, they mimicked the characteristics of ApoEVs to construct nanoparticles to address diseases. For example, Kraynak et al. coextruded plasma membranes from 3T3 fibroblasts, PS liposomes and poly (lactic-co-glycolic) acid nanoparticles to develop PS/membrane-coated nanoparticles and demonstrated them to be anti-inflammatory [21]. Toita et al. also constructed apoptotic-cell-mimicking PS-containing liposomes to enhance M1-to-M2 macrophage polarization, suppress the formation of pressure ulcers and facilitate their healing [79]. In the context of infectious diseases, asymmetric apoptotic body-like liposomes containing PS mimicking ApoBDs were generated to treat chronic P. aeruginosa infection [57]. Gramatica et al. designed PS-containing immunoliposomes to bind HIV-1 virus-like particles and proved that they could be internalized by macrophages, subsequently activating the immune system against HIV-1 and relieving persistent inflammation [80]. Hatakeyama et al. explored the function of PS-containing liposomes (PSLs) together with hydroxyapatite in bone defects in rats and demonstrated that PSLs can mimic the effect of apoptotic cells, regulate osteoblast differentiation, and thus promote bone regeneration [81].

Nevertheless, in most experiments, ApoEVs mimicking nanoparticles were not directly used; instead, they were constructed as delivery platforms to deliver drugs, nanoparticles, and small interfering RNAs (siRNAs), which will be explained in detail in the following sections.

#### Engineering apoptotic vesicles as carriers

EVs have been used as carriers for decades. They have a smaller size and lower immunogenicity than their parental cells [15] and have targeting abilities [15], a long circulation time and access to natural barriers [15, 16]. Among the investigated vesicles, exosomes have been widely explored for use as carriers because of their stable physicochemical properties, good biocompatibility, favourable cycling stability and low toxicity [82]. However, the separation and extraction of exosomes remains difficult and inefficient, which creates barriers to their production scale-up and clinical translation. The production of ApoBDs in apoptotic cells has a much higher efficiency [15, 16, 30]; however, they have not been utilized as carriers because of their varying size, complex contents, potential apoptosis-inducing activities and easy clearance by phagocytes [16]. Nevertheless, ApoEVs actually have many advantages when serving as carriers. In addition to their higher production efficiency, the production of ApoEVs is more easily controlled and scaled up, as cell apoptosis is better understood than the biogenesis process of exosomes or microvesicles [16]. Besides, the loading efficiency of molecules into ApoEVs is higher since molecules are packaged into ApoEVs automatically during apoptosis [16]. Additionally, because of the differences in biogenesis between exosomes and ApoEVs, cytomembrane-integrated protein molecules in ApoEVs are more abundant, which may be beneficial for targeted transport [16]. Furthermore, in many studies, ApoEVs with smaller sizes were stably isolated [16], while some methods were developed to reconstruct ApoEVs of smaller sizes and with better loading capabilities [60].

By inheriting surface proteins from parental cells, ApoEVs from immune cells and tumor cells retain homotypic affinity to target inflammation regions and tumors respectively [15, 60]. Moreover, due to the “find me” and “eat me” signals on their surface, ApoEVs display specific targeting abilities to phagocytes [15, 59]. Based on these characteristics, some studies have utilized ApoEVs as carriers. These studies can be classified into two categories: the first kind reconstructed ApoEVs derived from apoptotic cells, loaded them with the desired cargos and modified them to be more suitable as delivery platforms, while the second kind mimicked the surface structures of ApoEVs to construct nanoparticles.

##### Reconstructing apoptotic vesicles as carriers

When using ApoEVs as delivery platforms, loading cargos into vesicles is an important and challenging step. Some studies loaded the desired cargos into parental cells first and then induced cell apoptosis to produce ApoEVs with the desired cargos. By combining oligonucleotides (ASO) and a cationic konjac glucomannan, Wang et al. transfected ASOs into target cells and then treated them with H_2_O_2_ after ultraviolet (UV) radiation to induce apoptosis; this method proved to be simple, efficient and production-stable [16]. Via regulation by CD44V6, ApoBDs can be engulfed and released by ECs to cross the blood‒brain barrier [16]. When using this loading method, tumor cell-derived ApoBDs successfully delivered ASOs across the blood‒brain barrier and ameliorated Parkinson's disease in vivo, providing a potent strategy for delivering macromolecule drugs into the brain or crossing other physiological barriers by exploiting a natural route with good efficiency [16]. The ApoEVs isolated via this method were all smaller than 1000 nm in diameter [16]. Thus, they roughly classified ApoEVs into two kinds: large ApoEVs larger than 1 µm in diameter and small ApoEVs approximately 100–1000 nm in diameter [16]. Small ApoEVs contain no DNA fragments and are not easily engulfed, thus showing more potential to be used in drug delivery than typical ApoEVs [16]. Zheng et al. also loaded tumor cells with CpG immunoadjuvant-modified gold−silver nanorods (AuNR-CpG) through incubation first and induced them to undergo apoptosis later, which produced ApoEVs containing AuNR-CpG [83]. By making use of the ability of tumor cell-derived ApoEVs to target circulating monocytes and the tumor-homing behaviour of macrophages, a two-step targeting platform was established to accumulate nanomedicines in solid tumors, which can be used widely in tumor treatment to replace conventional tumor-targeted strategies due to its convenience and safety [83]. Specifically, Zhao et al. did not utilize or reconstruct ApoBDs directly; instead, they constructed PR104A-loaded nanoparticles to deliver medicines to external tumor cells and made use of the neighbour effect, which is mainly mediated by ApoEVs, to load antitumor medicines into endogenous ApoEVs induced by camptothecin and transport them from external apoptotic tumor cells to internal tumor cells [30]. In this way, drug penetration and whole-tumor destruction were enhanced in solid tumors [30].

**Fig. 2:**
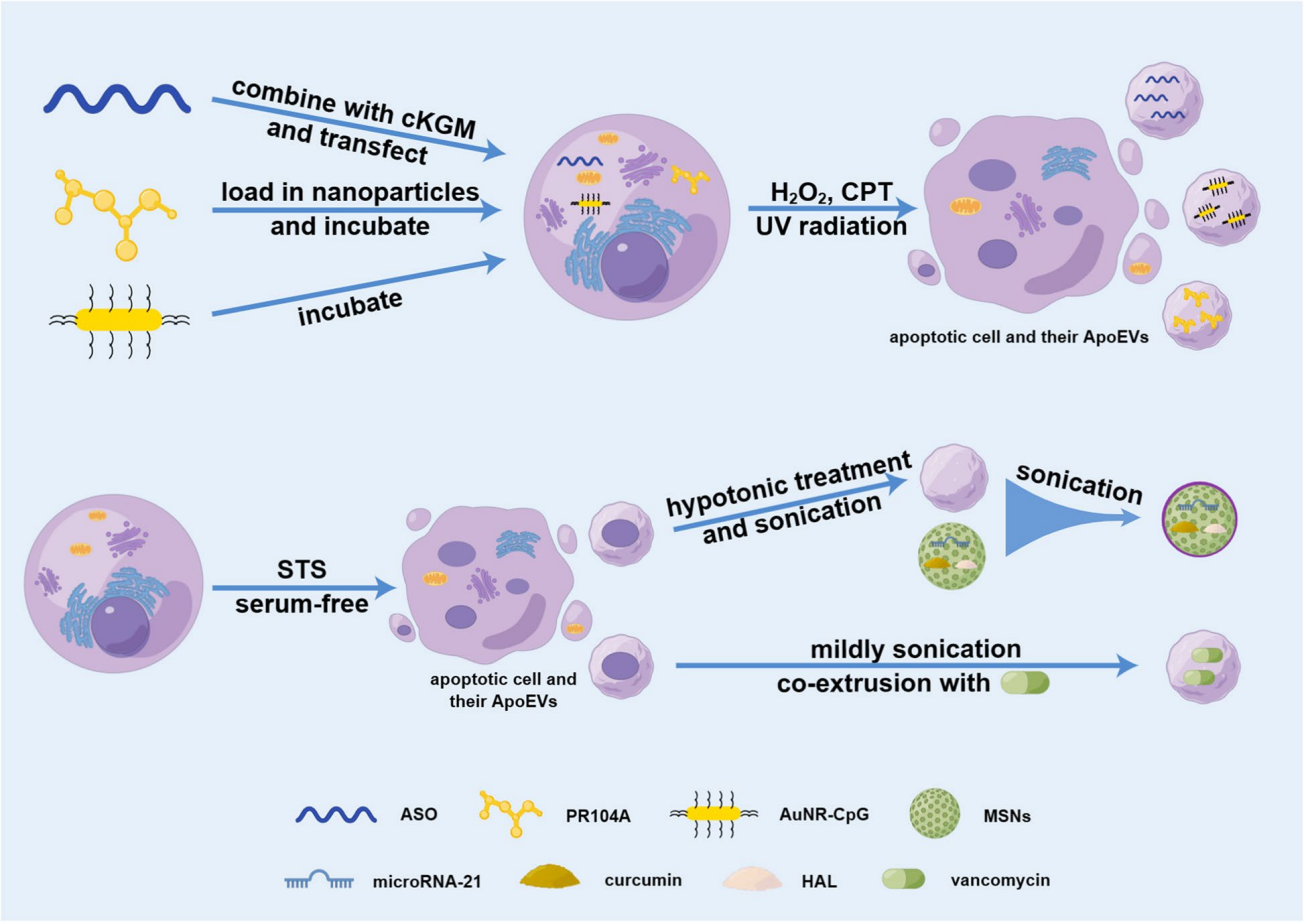
Engineering methods using ApoEVs as carriers. In some experiments, cells are first loaded with cargos and then induced to undergo apoptosis to produce ApoEVs with the cargos; in some experiments, cells are first induced to undergo apoptosis and then loaded with cargos or combined with cargo-preloaded MSNs. (Figure was created using Figdraw). Abbreviations: cKGM, cationic konjac glucomannan; CPT, camptothecin; UV, ultraviolet; STS, staurosporine; ASO, oligonucleotides; AuNR-CpG, CpG immunoadjuvant-modified gold−silver nanorods; MSNs, mesoporous silica nanoparticles; HAL, Hexyl 5-aminolevulinate hydrochloride

Also, some experts isolated ApoEVs first and then reconstructed and loaded cargos into them later. Dou et al. removed the residual components of ApoBDs from activated T cells through hypotonic treatment and sonication, and fused them with mesoporous silica nanoparticles (MSNs) through sonication, which were preloaded with the anti-inflammatory drug curcumin or microRNA, to construct chimeric apoptotic bodies [15]. By targeting and modulating the inflammatory abilities inherited from activated T cells, chimeric apoptotic bodies delivered molecules to inflamed regions, modulated inflammation and promoted regeneration [15]. This method proved to be successful in regulating cutaneous inflammation, facilitating regeneration, and improving inflammatory bowel diseases by modulating the macrophage phenotype [15]. Similarly, the components of ApoBDs from neutrophils were eliminated, and the pretreated ApoBDs were coextruded with MSNs to construct engineered neutrophil ApoBDs [72]. By inheriting surface signal molecules from parental cells, the engineered ApoBDs successfully transported hexyl 5-aminolevulinate hydrochloride to macrophages and improved inflammation in the context of myocardial infarction in vivo [72]. Additionally, Bose et al. extracted ApoEVs first and then sonicated them into small pieces and coextruded them together with vancomycin [60]. Through these processes, the size of the vesicles was largely reduced from 1−10 μm to approximately 100-150 nm, and the encapsulation efficiency of vancomycin was increased [60]. By making use of the abilities of ApoEVs derived from cancer cells to target macrophages and cancer cells, vancomycin was delivered to treat Staphylococcus aureus, which improved the effectiveness of treatment and decreased the adverse effects of vancomycin [60]. This approach can be further used to develop molecular therapies such as targeted nanocarriers to treat intracellular infections or tumors [60]. The engineering methods using ApoEVs as carriers are summarized (Fig. 2).

The above two loading strategies both have advantages and disadvantages. When loading cargos before inducing apoptosis, the ApoEVs produced may have less artificial biochemical conjugation; thus, undesirable changes to their structure and functions can be avoided [16]. However, with this method, it is difficult to load parental cells into cargos with high efficiency without impacting the structure, characteristics, and ApoEV production capabilities of the cells. By isolating ApoEVs and then reconstructing them, irrelevant and unwanted contents can be removed, which may increase the safety and standardization of engineered ApoEVs [15]. The difficulties of this method lie in how to encapsulate cargos into and modify ApoEVs without destroying the targeting or other capabilities of the ApoEVs. Investigations using ApoEVs as carriers are in the initial stage, and many issues deserve further and deeper exploration, including how to load cargos to ApoBDs effectively and efficiently, how to reconstruct standardized ApoEVs suitable for clinical translation, and the mechanism underlying the targeting abilities of ApoEVs. We also gathered detailed information on reconstructing ApoEVs as carriers to help solve these issues (Table 2).

**Table 2:** Applications of reconstructing apoptotic vesicles as carriers

Published year	Term	Parental cell	Size of vesicles	Induction method	Isolation method	Cargo	Function	Target cell	Animal	Applied method	Disease model	Ref.
2021	Apoptotic bodies	Rat bone marrow neutrophils	800-1200nm (Neu-ABs), 100-400nm (eNABs)	STS	Sequential centrifugation	HAL	Inflammation-tropism and immunoregulatory effects	Macrophages	Rats	Inject intravenously	Myocardial infarction	[72]
2021	Apoptotic bodies	External tumor cells	Approximately 950-1500nm	Nanoparticles loaded with anti-tumor drugs	Centrifugation	PR104A	Carry the remaining anti-tumor drugs to neighboring tumor cells	Internal tumor cells	Mice	Produced in vivo	Solid tumors	[30]
2021	Apoptotic bodies	B16F10	<800nm	H2O2 after UV radiation	Sequential centrifugation	ASO	Cross blood-brain barrier	Microglial cells	Mice	Inject intravenously	Parkinson's disease	[16]
2020	Apoptotic bodies	Mouse T cells	700-2000nm (ABs), 100-600nm (cABs)	STS	Sequential centrifugation	miR-21, curcumin	Target inflammatory regions and modulate inflammatory processes	Macrophages	Mice	Inject intravenously	Cutaneous inflammatory wound, colitis	[15]
2020	Apoptotic bodies	Mouse lymphoma cells	1-5μm	UV radiation	Sequential centrifugation	AuNR-CpG	Target circulating monocytes	Monocytes (monocytes then infiltrate the tumor center)	Mice	Inject intravenously	Solid tumors	[83]
2020	Reconstructed apoptotic bodies (ReApoBds)	Cancer cells	0.5 μm-10 μm (ApoBDs), 100-150nm (ReApoBds)	Serum starvation	Sequential centrifugation	Vancomycin	Target macrophages and cancer cells	Macrophages and cancer cells	Mice	Inject intravenously	Intracellular Staphylococcus aureus infection	[60]

*Abbreviations: Neu-Abs* apoptotic bodies from neutrophils, *eNABs* engineered neutrophil apoptotic bodies, *STS* Staurosporine, *HAL* Hexyl 5-aminolevulinate hydrochloride, *UV* Ultraviolet, *ASO* Oligonucleotides, *ABs* Apoptotic bodies, *cABs* chimeric apoptotic bodies, *AuNR-CpG* CpG immunoadjuvant-modified gold−silver nanorods, *ApoBDs* Apoptotic bodies, *ReApoBds* Reconstructed apoptotic bodies

##### Mimicking apoptotic vesicles as carriers

In recent decades, a great diversity of lipid nanoparticles was developed to serve as delivery platforms to transport drugs, molecules and RNA [84]. However, compared with artificial liposomes, vesicles derived from cells have natural advantages, including intercellular communication, recognition and targeting abilities, and responses to biologic signals [60]. Thus, lipid nanoparticles mimicking EVs are emerging to exploit the biological characteristics of EVs as well as the artificial features of nanoparticles. There are also liposomes mimicking ApoEVs, and most use PS to mimic the surface of ApoEVs.

Liposomes mimicking PS have been constructed as carriers to address various disease models, such as inflammation-related diseases, tumors and infectious diseases. To stabilize atherosclerotic plaques, apoptotic body biomimicking liposomes were constructed to selectively deliver pioglitazone, a peroxisome proliferator-activated receptor γ agonist, into atherosclerotic macrophages and upregulate anti-inflammatory macrophages while minimizing side effects [59]. To treat type 1 diabetes, insulin peptide-loaded PS-liposomes were used to induce immune tolerance [85, 86]. To treat tumors, Yin et al. designed matrix metalloproteinase 2-sensitive PS-modified nanoparticles to deliver the anticancer drug dasatinib for tumor-associated macrophage targeting and depletion as a new strategy and proved its accuracy and efficiency [20]. Some experts have entrapped drugs treating Leishmania into PS liposomes and proved their improved effect against Leishmania-infected macrophages [87, 88]. Besides, ApoEV-mimicking liposomes were used to deliver siRNA to macrophages to achieve knockdown of genes, which can overcome the difficulties of transporting siRNA to immune cells [89]. If this challenge can be overcome, this approach will probably surpass the use of viral vectors in gene transfection due to the potential risks of viruses [89].

In conclusion, by mimicking PS on the surface of ApoEVs, nanoparticles can interact with macrophages or other phagocytes, thus delivering molecules to recipient cells. In addition to the strategies utilized above, more potential usages can be developed on the basis of this theory, for example, mimicking other “eat me” signals during efferocytosis or investigating phagocyte-surface receptors that recognize PS.

#### Constructing apoptotic vesicles as vaccines

##### Vaccines for autoimmune diseases

**Fig. 3:**
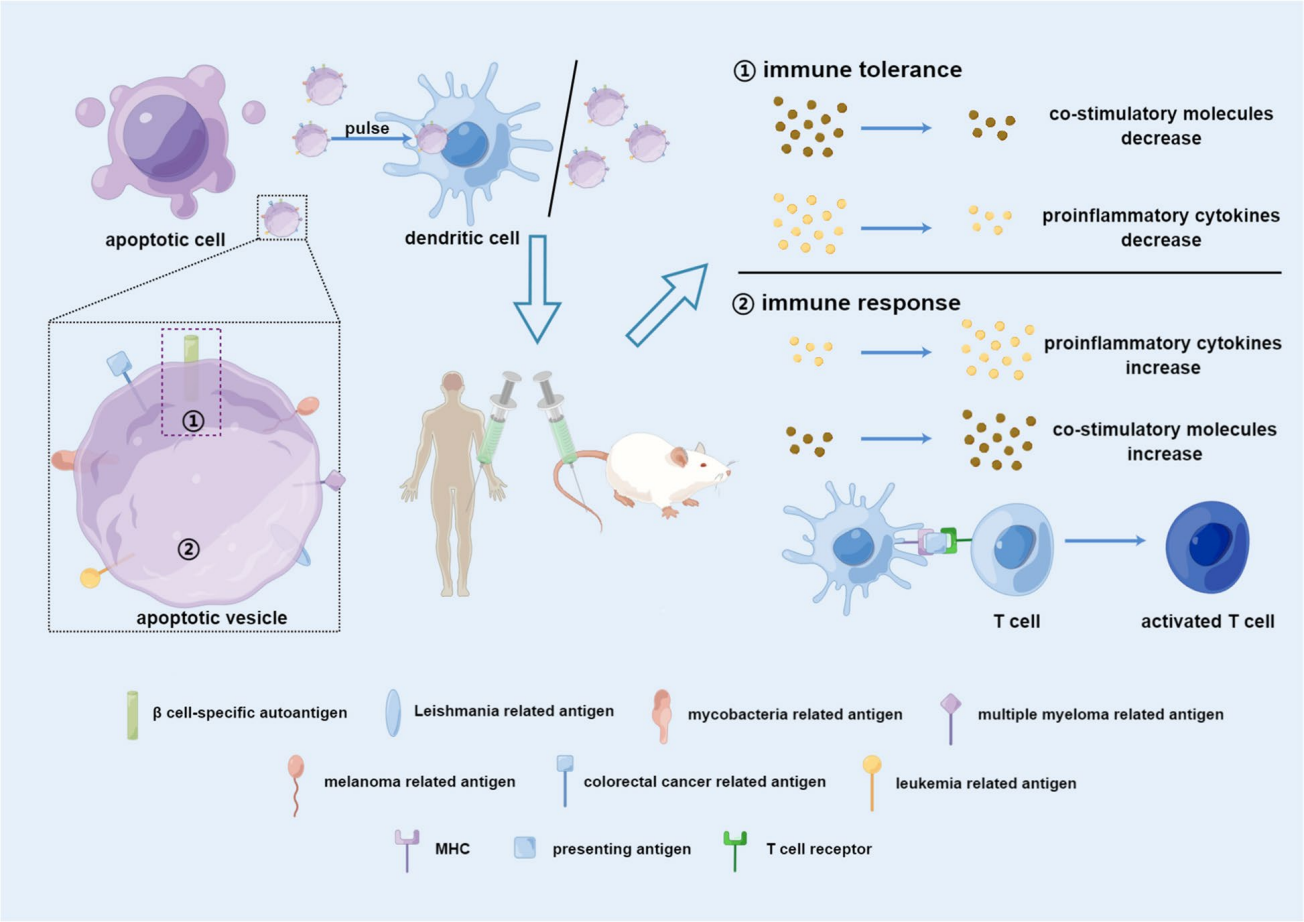
Using ApoEVs as vaccines. ApoEVs can inherit antigens from parental cells, including autoantigens, antigens from pathogens and tumor cells. Thus, ApoEVs can be a source of antigens in vaccines. When injected into human beings or animals directly or after being loaded into DCs, they can either enhance immune tolerance or promote an immune response dependent on the transported antigens. (Figure was created using Figdraw). Abbreviations: MHC, major histocompatibility complex

During the early stages of apoptosis, autoantigens are translocated into ApoEVs [90]. For example, SLE antigens, including nucleosomal DNA and nuclear ribonucleoproteins, are found in ApoBDs [91]. Vitiligo autoantigens can also translocate into ApoBDs, which are regulated by the cytoskeletal protein activation pathway and the c-Jun N-terminal kinase-related apoptosis pathway [92]. In addition, after asbestos induction, apoptotic cell surface blebs rich in SSA/Ro52, a kind of autoantigen, can be formed in murine macrophages [93]. In ApoBDs from thymus cells of mice, autoantigens related to human autoimmune diseases can also be found [94]. The above studies proved that ApoEVs can inherit autoantigens from their parental cells, which provides a theoretical basis for the possibility of using ApoEVs as vaccines for autoimmune diseases (Fig. 3 and Table 3).

**Table 3:** Applications of constructing apoptotic vesicles as vaccines

Published year	Term	Parental cell	Apoptosis induction method	Type of experiment	Animal	Applied method	Disease	Duration (clinical trials only)	Sample capacity (clinical trials only)	Ref.
2020	Apoptotic blebs	Macrophages J774A.1	The Apoptosis Inducer Kit	Animal experiment	Mice	Inject subcutaneously	Cutaneous leishmaniasis	N/A	N/A	[95]
2019	Apoptotic vesicles	Human melanoma cells	Bortezomib	Cell experiment	N/A	N/A	Malignant melanoma	N/A	N/A	[96]
2014	Apoptotic blebs	HL-60	Heat shock	Cell experiment	N/A	N/A	Myeloid leukemia	N/A	N/A	[97]
2014	Apoptotic blebs	HL-60	Heat shock	Cell experiment	N/A	N/A	Acute myeloid leukemia	N/A	N/A	[98]
2012	Apoptotic bodies	Chronic lymphocytic leukemia cells	Irradiation	Clinical trial	N/A	N/A	Chronic lymphocytic leukemia	52 weeks	15	[99]
2012	Apoptotic vesicles	Macrophages	Free of serum or BCG infection	Cell and animal experiment	Mice	Inject subcutaneously	Tuberculosis	N/A	N/A	[100]
2010	Apoptotic bodies	β cells	UVB irradiation	Cell and animal experiment	Mice	Inject intraperitoneally	Type 1 diabetes	N/A	N/A	[101]
2010	Apoptotic bodies	ARH77 myeloma cells	Irradiation	Cell experiment	N/A	N/A	Multiple myeloma	N/A	N/A	[102]
2009	Apoptotic bodies	Human melanoma cells	Dactinomycin	Clinical trial	N/A	N/A	Malignant melanoma	70 months	9	[103]
2006	Apoptotic vesicles	Macrophages	Free of serum	Cell and animal experiment	Mice	Inject subcutaneously	Tuberculosis	N/A	N/A	[35]
2005	Apoptotic bodies	Human leukemic B cells	Gamma ray	Clinical trial	N/A	N/A	B cell chronic lymphocytic leukemia	Exceed 23 months	9	[104]
2004	Apoptotic bodies	Chronic lymphocytic leukemia cells	Serum-free and four freeze–thaw cycles	Cell experiment	N/A	N/A	B cell chronic lymphocytic leukemia	N/A	N/A	[105]
2002	Apoptotic bodies	Melanoma cells	UVB irradiation, arginine butyrate or heat shock	Cell experiment	N/A	N/A	N/A	N/A	N/A	[106]
1997	Apoptotic bodies	Rat colon carcinoma cells	Sodium butyrate	Animal experiment	Rat	Inject subcutaneously or intraperitoneally	Colorectal cancer	N/A	N/A	[107]

*Abbreviations: BCG* Bacillus Calmette-Guerin

As a practical example, DCs pulsed with antigen-specific ApoBDs from beta cells could reduce the expression of the costimulatory molecules CD40 and CD86 and the secretion of the proinflammatory cytokines interleukin (IL) 6 and tumor necrosis factor α (TNF α), while significantly reducing the diabetes incidence in vivo, proving to be a promising method to prevent type 1 diabetes [101]. In that study, the ApoBDs were not isolated and characterized; instead, beta cells were induced to undergo apoptosis by ultraviolet radiation β irradiation and cocultured with DCs [101]. Thus, to be more precise, antigen-specific apoptotic cells were loaded into DCs to exert a type 1 diabetes-preventive effect [101]. If the product of apoptotic cells can be identified and characterized, the preventive effect may be more stable and replicable. However, this approach still provides strategies for the prevention or remission of type 1 diabetes and other autoimmune diseases.

##### Vaccines for infectious diseases

ApoEVs can serve as transport media for pathogens of various kinds of infectious diseases. HIV-1 DNA can be transported to recipient cells through ApoBDs without the need for CD4 [108]. Moreover, ApoBDs from Epstein‒Barr virus (EBV)-carrying B lymphocytes can transfer EBV to cells lacking receptors for EBV [109]. In addition, ApoBDs from influenza A virus (IAV)-infected monocytes, which contain IAV mRNA, protein and virions, can facilitate viral replication in vitro and in vivo [110]. Apoptotic blebs containing Chikungunya virus, a virus spread by mosquito bites, can infect macrophages without causing inflammation, which may be a mechanism by which viruses infect host cells while escaping the immune response of host cells [111].

As ApoEVs are closely related to cell- pathogen communication and immune responses in infectious diseases, they also play an important role in vaccines for different kinds of pathogens (Fig. 3 and Table 3), which are safer than the infected cells themselves. They can transport mycobacterial antigens from infected macrophages to DCs, subsequently activating CD8^+^ T cells, which demonstrates that vaccines with ApoEVs from infected cells can protect against *Mycobacterium tuberculosis* [35]. Moreover, ApoEVs from recombinant tuberculosis vaccine (recombinant Bacillus Calmette-Guerin, rBCG)-infected macrophages better primed CD4^+^ and CD8^+^ T cells, which may contribute to the improved efficacy of rBCG compared to parental BCG [100]. Faridnia et al. proved the efficacy of apoptotic blebs from *Leishmania major*-infected macrophages against *Leishmania major* in vivo, with increases in IFN γ levels and the lymphocyte proliferation index [95].

##### Vaccines for tumors

Phagocytosis of ApoBDs derived from oncogene-transfected fibroblasts resulted in changes in tumor cell behaviour in vitro and in vivo, which supported horizontal transfer of oncogenes via the uptake of ApoBDs [112]. As ApoEVs can inherit tumor-related substances from their parental cells, they can be utilized as a source of antigens in tumor vaccines (Table 3 and Fig. 3). Cancer immunotherapy has unique advantages, including enhancing the immune response, being suitable for various kinds of cancers, and having lasting effects [113]. The history of ApoEVs as tumor vaccines can be traced back to several decades ago.

Early in 1997, Boisteau et al. proved that treatment with IL 2 as well as ApoBDs induced by sodium butyrate could obviously improve colorectal cancer remission and survival rates in vivo [107]. The mice immunized with ApoBDs gained enduring immunity, and antibodies to tumor cells and ApoBDs were generated in the serum [107]. In another report, compared with peptide-pulsed DCs, ApoBD-loaded DCs could cross-prime T cells specific for the NA17-A antigen but not for the Melan-A/MART-1 antigen, probably because of the preservation or alteration of antigen expression [106]. This means that if antigens can be inherited by ApoBDs, ApoBDs can be utilized in tumor immunotherapies. Kokhaei et al. compared the function of ApoBDs, tumor lysates, and tumor RNA when delivering antigens to DCs and proved the superiority of cellular vaccines containing DCs loaded with ApoBDs in B-cell chronic lymphocytic leukaemia, as they increased the secretion of proinflammatory cytokines and costimulatory molecules and decreased the secretion of anti-inflammatory cytokines [105]. The ability of ApoBDs to take up and process more antigens may contribute to this result [105]. Ruben et al. compared apoptotic blebs and apoptotic cell remnants and concluded that apoptotic blebs are more suitable for tumor vaccines, with higher production of IFN γ [98]. They also proved the efficiency of dermally applied vaccines by using apoptotic blebs [97]. Intradermally administered apoptotic blebs can be engulfed by mature skin DC subsets, cross-present tumor-associated antigens, increase the levels of costimulatory molecules, and prime effector T cells [97]. In addition, by modifying ApoEVs with high-mannose glycans, ApoEVs are more likely to be engulfed, and thus, they are more able to prime tumor-specific CD8^+^ T cells [96]. High-mannose glycans are ligands of dendritic cell-specific intercellular adhesion molecule-3-grabbing nonintegrin, which is a DC-associated C-type lectin receptor that can promote engulfment [96]. Additionally, Ocadlikova et al. compared the efficiency of tumor-associated peptides and ApoBDs when used in DC vaccines and found that they could both lead to myeloma-specific T-cell responses in vitro [102].

In a clinical trial, immunotherapies with allogeneic DCs pulsed with tumor lysates or ApoBDs were tested in nine patients with B-cell chronic lymphocytic leukaemia [104]. After vaccination, the numbers of leukaemic cells in most patients decreased, the levels of proinflammatory factors, including TNF α, INF γ, and IL 2, increased, and immunotherapy was proven safe, available and tolerated [104]. Moreover, Palma et al. constructed Apo-DCs as a vaccine for chronic lymphocytic leukaemia patients and proved it to be efficient and safe [99]. The addition of adjuvants such as granulocyte–macrophage-colony-stimulating-factor and low-dose cyclophosphamide could further enhance the immunogenicity of the vaccine [99]. However, in a clinical trial, melanoma apoptotic body-pulsed DCs were found to have limited efficiency and should be combined with other treatments [103].

An ideal vaccine should be safe while inducing lasting immunoreactivity of T cells [95]. The effect of immunotherapy is highly related to the immunogenicity of the cells, while some tumor cells have relatively low immunogenicity, making immunotherapy rather difficult. Major studies on tumor vaccines utilized a single antigen, while vaccines with multiple antigens can broaden the immune response and avoid immune escape [96]. ApoEVs pulsed with DCs are more immunogenic and are not dependent on one particular type of antigen [114]. When using ApoEVs as vaccines or in immunotherapy, two major approaches have been used: pulsing them into DCs to utilize them as a source of antigens in DC vaccines or directly injecting them to activate DCs in vivo.

Although ApoEVs can transport antigens and serve as vaccines, few studies have been conducted in this field. This may be due to the varying sizes and complicated contents of ApoEVs, which may significantly influence their exact effects. Compared with exosomes and microvesicles, which are well characterized, many properties of ApoEVs are unknown [115]. For example, in some studies, ApoEVs could promote the maturation of DCs, while in other studies, ApoEVs did not have this function, which may be because of the heterogeneity of the ApoEVs used in various studies [96]. In addition, Horrevorts et al. found that ApoEVs derived from murine ECs 1-3 μm in diameter contained IL 1α, while those smaller than 1 μm did not, indicating the influence of size on ApoEVs [96]. Additionally, the terminology of ApoEVs is not standardized, and many terms used in articles did not refer to the exact subtypes of ApoEVs while some were even corpses instead of vesicles. Thus, only with a deeper understanding of the formation mechanism and content distribution of ApoEVs, as well as standardized terminology and isolation methods, can the usage of ApoEVs in vaccines be more effective and accessible.

#### Diagnostic applications of apoptotic vesicles

As an important medium of intercellular communication, EVs contain many signalling molecules that can be used in diagnostic applications. Exosomes have been widely explored as biomarkers to diagnose various kinds of diseases, including cancers, cardiovascular diseases, and neurodegenerative diseases, and to predict prognosis [116, 117]. There have also been some studies on the diagnostic function of ApoEVs.

First, ApoEVs can be used in the diagnosis of malignant tumors. Eerola et al. demonstrated that compared with the presence of malignant cells, the presence of alveolar macrophages with ApoBDs in sputum smears could be a more sensitive marker of pulmonary malignancy, indicating their potential in detecting lung carcinoma [118]. Additionally, ApoBDs were proven to be a uniform morphologic characteristic of endocervical adenocarcinoma in situ [119], and Brustmann et al. found that ApoBDs are a marker for classifying serous ovarian carcinomas [120]. Aihara et al. reported that ApoBDs and the Gleason grade of carcinoma of the prostate appeared to be positively correlated, which means that ApoEVs have the potential to predict the prognosis of carcinoma of the prostate to some extent [121], while Aydin et al. held the opinion that the existence of ApoBDs should also be examined when diagnosing difficult cases of prostate cancer by needle biopsy [122]. Lázaro-Ibáñez et al. demonstrated the differences in gDNA among three kinds of EVs from prostate cancer cells and possible specific mutations, which has the potential to be used to diagnose cancer and predict its prognosis [123].

Besides, ApoEVs are also utilized in the diagnosis of the digestive system. Crypt apoptosis is an important diagnostic criterion of graft versus host disease (GVHD) but can also be present in other conditions [124]. Lin et al. discovered that the presence of six or fewer crypt ApoBDs in colon biopsies may indicate the probability of GVHD but is not sufficient for diagnosis [124]. Crypt apoptosis is also a widely used criterion in the diagnosis of acute cellular rejection (ACR) [125]. Moreover, apoptosis of T cells and engulfment of ApoBDs can also be utilized in diagnosing ACR against intestinal allografts [125]. Although the count of ApoBDs is considered a diagnostic criterion for GVHD and ACR, merely utilizing this index may not be sufficient and cannot accurately distinguish these diseases from the normal ileal mucosa [126]. Crypt ApoBDs can also be used to predict villous atrophy recovery after gluten-free diet treatment in celiac disease [127, 128]. Furthermore, the count of portal ApoBDs can be used for the diagnosis of active autoimmune hepatitis, particularly in early biopsies when no other conventional characteristics can be found [129].

Also, ApoEVs from blood samples may help monitor cerebrovascular and neurodegenerative diseases, and this approach is highly efficient and causes little injury [130]. Evidence of ApoEVs in autoimmune diseases has also been found. Koopman et al. found nuclear proteins and ApoBDs in the lupus band of patients with cutaneous lupus erythematosus, which can potentially be used in the diagnosis of cutaneous lupus erythematosus [131].

Finally, ApoEVs can also be used in imaging. By mimicking ApoEVs, PS has been used in molecular imaging approaches to target and image macrophages to predict the plaque vulnerability of atherosclerotic lesions [132, 133] and it has also been utilized for magnetic resonance imaging and confocal microscopy imaging of macrophages [134].

## Conclusion and further perspectives

Exosomes are the most well-studied extracellular vesicles. ApoEVs and exosomes show notable differences in size, biogenesis, molecular markers and characteristics [29]. The size of exosomes mainly ranges from 30 to 150 nm, whereas that of ApoEVs has a greater range [25, 135]. For biogenesis, exosomes are generated when multivesicular bodies fuse with the plasma membrane and are released [136], which is different from ApoEVs. Tsg101, Alix, CD81, CD82, CD63, and CD9 are conventional exosomal markers [137], whereas ApoEVs express the specific markers caspase 3, calreticulin, S1PR1, and Annexin V [62, 63, 68]. Studies on the applications of ApoEVs have increased following a deeper understanding of their composition and characteristics. ApoEVs indeed have superiority in some contexts. In inflammation-related diseases or conditions, the phagocytosis of apoptotic cells contributes to immune tolerance and enhances tissue regeneration [72]. However, administration of apoptotic debris or apoptotic cells can lead to secondary infection of macrophages or autoimmunity [72]. Thus, by inheriting important molecules and properties from their parental cells, the administration of ApoEVs might be a great alternative treatment [72]. Compared with microvesicles and exosomes, ApoEVs may be more suitable to modulate inflammation and promote tissue regeneration in inflammatory contexts [15, 72]. Similarly, ApoEVs are quite suitable for reconstruction as delivery platforms targeting inflamed sites or immune cells.

In addition, the function of ApoEVs in ischaemic and anoxic environments, such as the dental pulp cavity and solid tumors, deserves deeper exploration, as cells tend to undergo apoptosis in such environments. Additionally, apoptosis has been regarded to promote regeneration [138], and ApoEVs also play an important role in MSC transplantation and have been proven to be useful in the regeneration of various kinds of tissues. And the effects of ApoEVs from other kinds of cells during tissue regeneration need further investigation.

However, extracellular vesicles can lead to a hypercoagulable state [139]. ApoEVs from melanoma cells are more likely to cause coagulation than exosomes [140] while ApoEVs from tumors exhibit better procoagulant activity than their parental cells [141]. Tumor-derived apoptotic vesicles have procoagulant activity mainly dependent on tissue factors and PS [142], and PS can also contribute to their immunogenicity [143]. ApoEVs derived from tumor increase the incidence rates of venous thromboembolism in cancer patients especially those receiving chemotherapy [143]. Thus, when directly using ApoEVs or engineering them as carriers, it is necessary to consider their safety and immunogenicity problems. When conducting animal experiments and further clinical trials, coagulation indicators should be detected. Also, with more in-depth studies of their procoagulation mechanism, it may be possible to modify their surface molecules to vary their procoagulant property. Moreover, their procoagulant activity may be developed to be used in haemorrhagic disorders.

Another critical barrier when translating EVs to clinical use is their heterogeneity, and this issue is particularly prominent for ApoEVs. The heterogeneity of ApoEVs can be observed from multiple perspectives, including their size, surface molecules, contents and properties, which can all affect their functions. For example, size can influence their ability to cross biological barriers, surface molecules can impact their targeting and ingestion capabilities, and their contents can affect their roles in regulating inflammation and promoting regeneration.

The properties and functions of ApoEVs can be influenced by multiple factors, including the parental cell size and type, the formation mechanism, induction and isolation methods, and reconstruction and preservation methods [15]. By summarizing the direct applications of ApoEVs and applications using ApoEVs as carriers, we found that most experiments induce parental cells to undergo apoptosis with STS; other methods include serum-free treatment and UV irradiation (Tables 1, 2 and 3). Some studies have already compared the effects of different apoptosis induction methods on ApoEV functions, but more experiments are needed to explore this area. In most cases, ApoEVs were isolated by gradient centrifugation methods, although the exact rotation speeds and times differed across experiments. The sizes of ApoEVs vary from tens of nanometres to thousands of nanometres (Tables 1 and 2). Besides, although the isolated vesicles were named ApoBDs in some studies, their sizes were far smaller than 1 μm. The naming rules of ApoEVs need to be uniform to make relevant studies more standardized. This relies on a better understanding of the differences among subtypes of ApoEVs.

The process of apoptosis and the formation of ApoEVs varies and is not standardized, especially when cargos are loaded into them. Membrane molecules and contents inherited from source cells play a significant role in the properties and functions of ApoEVs. However, as EVs selectively choose cargos from source cells, which is also influenced by the surrounding microenvironment during biogenesis [41], there are differences in substances between ApoEVs and their parental cells [11]. During the formation of exogenous and endogenous ApoEVs, the mechanism by which ApoEVs inherit these molecules remains unknown. Thus, to translate exogenous ApoEVs for scaled-up production and clinical use, donor cell culture conditions, as well as the isolation and characterization of ApoEVs, need to be standardized [15]. Similar to exosomes and microvesicles, which also face these challenges, the standardization of ApoEVs should be performed according to an authoritative standard such as the Minimal Information for Studies of Extracellular Vesicles (MISEV) guidelines [144]. The MISEV guidelines were developed in 2014 and revised in 2018, and they summarize in detail the collection and preprocessing process of all types of sample sources, the separation, concentration, and characterization process of extracellular vesicles, and methods for conducting functional studies of extracellular vesicles [144]. Using the MISEV guidelines, studies on extracellular vesicles can be more reliable and reproducible [144]. In addition, after administration of exogenous ApoEVs, their distribution and metabolism significantly influence their desired effects and side effects [72]; thus, the distribution and metabolism of ApoEVs require further investigation [15].

To date, most studies on ApoEVs have concentrated on the function of exogenous ApoEVs. However, the applications of exogenous ApoEVs face issues such as immunogenicity, safety, heterogeneity and restricted sources. Therefore, it is necessary to investigate the source and distribution of endogenous ApoEVs. However, it is obvious that issues related to endogenous ApoEVs require complicated experimental conditions and are challenging to explore. Generally, although there is still a long way to go in the investigation of ApoEVs, they are worthy of further study and have application potential.

## Data Availability

Not applicable.

## Abbreviations

EVs: Extracellular vesicles
ApoEVs: Apoptotic vesicles
ApoBDs: Apoptotic bodies
ApoMVs: Apoptotic microvesicles
ApoExos: Apoptotic exosomes
S1P: Sphingosine-1-phosphate
PS: Phosphatidylserine
ECs: Endothelial cells
DCs: Dendritic cells
IFN: Interferon
TCTP: Translationally controlled tumor protein
S1PR: Sphingosine-1-phosphate receptors
DAMPs: Damage-associated patterns
ADP: Adenosine diphosphate
MLCK: Myosin light chain kinase
PANX1: Pannexin 1
ROCK1: Rho-associated protein kinase 1
LIMK1: LIM domain kinase 1
PAK2: p21-activated kinase
ADSCs: Adipose mesenchymal stem cells
STS: Staurosporine
miR: microRNA
ESCs: Embryonic stem cells
UCMSCs: Umbilical cord mesenchymal stem cells
iPSCs: induced pluripotent stem cells
MSCs: Mesenchymal stem cells
BMMSCs: Bone marrow mesenchymal stem cells
TFEB: transcription factor EB
hDPSCs: human dental pulp cells
sEVs: small extracellular vesicles
FasL: Fas ligand
ROS: reactive oxygen species
mOCs: mature osteoclasts
pOC-ABs: preosteoclast apoptotic bodies
mOC-ABs: mature osteoclast apoptotic bodies
EPCs: endothelial progenitor cells
PSLs: Phosphatidylserine-containing liposomes
siRNA: small interfering RNA
ASO: oligonucleotides
UV: Ultraviolet
AuNR-CpG: CpG immunoadjuvant-modified gold−silver nanorods
MSNs: Mesoporous silica nanoparticles
TNBC: Triple-negative breast cancer
IL: Interleukin
TNF α: Tumor necrosis factor α
EBV: Epstein-Barr virus
IAV: Influenza A virus
BCG: Bacillus Calmette-Guerin
GVHD: Graft versus host disease
ACR: Acute cellular rejection
